# Pemetrexed Enhances Membrane PD-L1 Expression and Potentiates T Cell-Mediated Cytotoxicity by Anti-PD-L1 Antibody Therapy in Non-Small-Cell Lung Cancer

**DOI:** 10.3390/cancers12030666

**Published:** 2020-03-12

**Authors:** Andrea Cavazzoni, Graziana Digiacomo, Roberta Alfieri, Silvia La Monica, Claudia Fumarola, Maricla Galetti, Mara Bonelli, Daniele Cretella, Valeria Barili, Alessandra Zecca, Elisa Giovannetti, Michelangelo Fiorentino, Marcello Tiseo, Pier Giorgio Petronini, Andrea Ardizzoni

**Affiliations:** 1 Department of Medicine and Surgery, University of Parma, 43126 Parma, Italy; graziana.digiacomo@unipr.it (G.D.); silvia.lamonica@unipr.it (S.L.M.); claudia.fumarola@unipr.it (C.F.); mara.bonelli@unipr.it (M.B.); daniele.cretella@unipr.it (D.C.); barili.valeria@gmail.com (V.B.); marcello.tiseo@unipr.it (M.T.); piergiorgio.petronini@unipr.it (P.G.P.); 2 Italian Workers’ Compensation Authority (INAIL) Research Center, 43126 Parma, Italy; m.galetti@inail.it; 3 Department of Infectious Diseases and Hepatology, University Hospital of Parma, 43126 Parma, Italy; alessandrazecca.az@gmail.com; 4 Department of Medical Oncology, Cancer Center Amsterdam, Amsterdam University Medical Center, 1081HV Amsterdam, The Netherlands; elisa.giovannetti@gmail.com; 5 Fondazione Pisana per la Scienza, San Giuliano Terme, 56017 Pisa, Italy; 6 Department of Experimental, Diagnostic and Specialty Medicine, University of Bologna, 40138 Bologna, Italy; michelangelo.fiorentino@unibo.it (M.F.); andrea.ardizzoni2@unibo.it (A.A.)

**Keywords:** pemetrexed, PD-L1, IFN-γ, NSCLC, chemotherapy

## Abstract

Immunotherapy has significantly changed the treatment landscape for advanced non-small-cell lung cancer (NSCLC) with the introduction of drugs targeting programmed cell death protein-1 (PD-1) and programmed cell death ligand-1 (PD-L1). In particular, the addition of the anti-PD-1 antibody pembrolizumab to platinum-pemetrexed chemotherapy resulted in a significantly improved overall survival in patients with non-squamous NSCLC, regardless of PD-L1 expression. In this preclinical study, we investigated whether chemotherapy can modulate PD-L1 expression in non-squamous NSCLC cell lines, thus potentially affecting immunotherapy efficacy. Among different chemotherapeutic agents tested, only pemetrexed increased PD-L1 levels by activating both mTOR/P70S6K and STAT3 pathways. Moreover, it also induced the secretion of cytokines, such as IFN-γ and IL-2, by activated peripheral blood mononuclear cells PBMCs that further stimulated the expression of PD-L1 on tumor cells, as demonstrated in a co-culture system. The anti-PD-1/PD-L1 therapy enhanced T cell-mediated cytotoxicity of NSCLC cells treated with pemetrexed and expressing high levels of PD-L1 in comparison with untreated cells. These data may explain the positive results obtained with pemetrexed-based chemotherapy combined with pembrolizumab in PD-L1-negative NSCLC and can support pemetrexed as one of the preferable chemotherapy partners for immunochemotherapy combination regimens.

## 1. Introduction

Programmed cell death protein-1 (PD-1), a member of the CD28 family of proteins, is mainly expressed on T-cell surface [1] and its ligand, programmed cell death ligand-1 (PD-L1), is frequently overexpressed in many types of human cancers, including non-small-cell lung cancer (NSCLC), and is associated with a poor prognosis [2]. PD-1/PD-L1 axis is a key immune checkpoint pathway that provides inhibitory signals to the immune system in order to modulate the activity of T cells and, therefore, plays a crucial role in cancer progression by altering the status of immune surveillance. The engagement of PD-1 by PD-L1 induces an inhibitory signal resulting in the reduction of T-cell proliferation and cytotoxic activity. The cytotoxic properties of T cells can be restored by blocking this interaction with specific drugs (anti-PD-1/PD-L1 monoclonal antibodies) [3].

Recently, immune checkpoint inhibitors have revolutionized the medical treatment approach for metastatic NSCLC patients without oncogenic druggable alterations. The combination of these agents with chemotherapy, both in non-squamous [4] and squamous [5] histologies, has resulted in a significant overall survival (OS) benefit in comparison to standard chemotherapy. In particular, the results of the Keynote-189 [4] phase III clinical trial showed that pembrolizumab added to platinum-pemetrexed doublet led to a 50% mortality reduction when compared with chemotherapy alone.

Of note, the survival benefit observed for pemetrexed based-chemotherapy combined with pembrolizumab was achieved regardless of PD-L1 expression at baseline and it was statistically significant and clinically relevant even in patients with PD-L1-negative tumors. This finding differs from that observed using immunotherapy as a single treatment, whose efficacy appears to be driven, at least in part, by PD-L1 expression [6].

Emerging data seem to support an effect of chemotherapy on PD-L1 expression in NSCLC [7,8] and increasing evidence indicates that PD-L1 expression may be regulated by both intrinsic and extrinsic mechanisms in different tumor types [9,10]. The presence of oncogenic drivers, such as *EML4-ALK* rearrangement or *EGFR* activating mutations, in NSCLC patients can also cause an increase in PD-L1 level [11,12] and treatment with specific ALK or EGFR inhibitors has been shown to reduce this expression [11]. Similarly, *PTEN* loss or *PIK3CA* mutations were shown to activate the AKT/mTOR pathway with subsequent increase of PD-L1 expression in melanoma and NSCLC [13] and treatment with specific PI3K inhibitors caused a reduction of PD-L1 expression [14].

An extrinsic upregulation of PD-L1 in cancer cells is also dependent on IFN-γ-mediated signaling pathway. IFN-γ, once bound to a member of the IFNGR1-2 receptor family, activates JAK/STAT intracellular signaling with the induction of interferon-regulated factor-1 (IRF-1), which is the main factor responsible for PD-L1 expression [10].

Previous studies showed that several anticancer drugs can modulate PD-L1 expression in different cancer cell lines. For instance, an increase in PD-L1 has been described in breast cancer cells after treatment with paclitaxel, etoposide, 5-fluorouracil (5-FU) [15], and irinotecan [16]; gemcitabine or paclitaxel resulted in enhanced expression of PD-L1 in ovarian cancer cell lines in an NF-kB-dependent manner [17], while carboplatin plus paclitaxel or 5-FU plus cisplatin led to an increase of PD-L1 expression in esophageal squamous cell carcinoma [18].

The aim of the present study was to evaluate the effects of standard chemotherapeutic drugs on the modulation of PD-L1 expression in non-squamous *EGFR* and *ALK* wild-type NSCLC cell lines. To our knowledge, this is the first demonstration that pemetrexed increases PD-L1 levels by activating both mTOR/P70S6K and STAT pathways in this type of cancer cells. Moreover, pemetrexed increased the secretion of cytokines, such as IFN-γ and IL-2, which stimulated a further increase in PD-L1 expression on tumor cells in a co-culture system and promoted T cell-mediated cytotoxicity when associated with atezolizumab.

## 2. Results

### 2.1. Pemetrexed Induces the Expression of PD-L1 in Human Adenocarcinoma NSCLC Cell Lines

**Figure 1:**
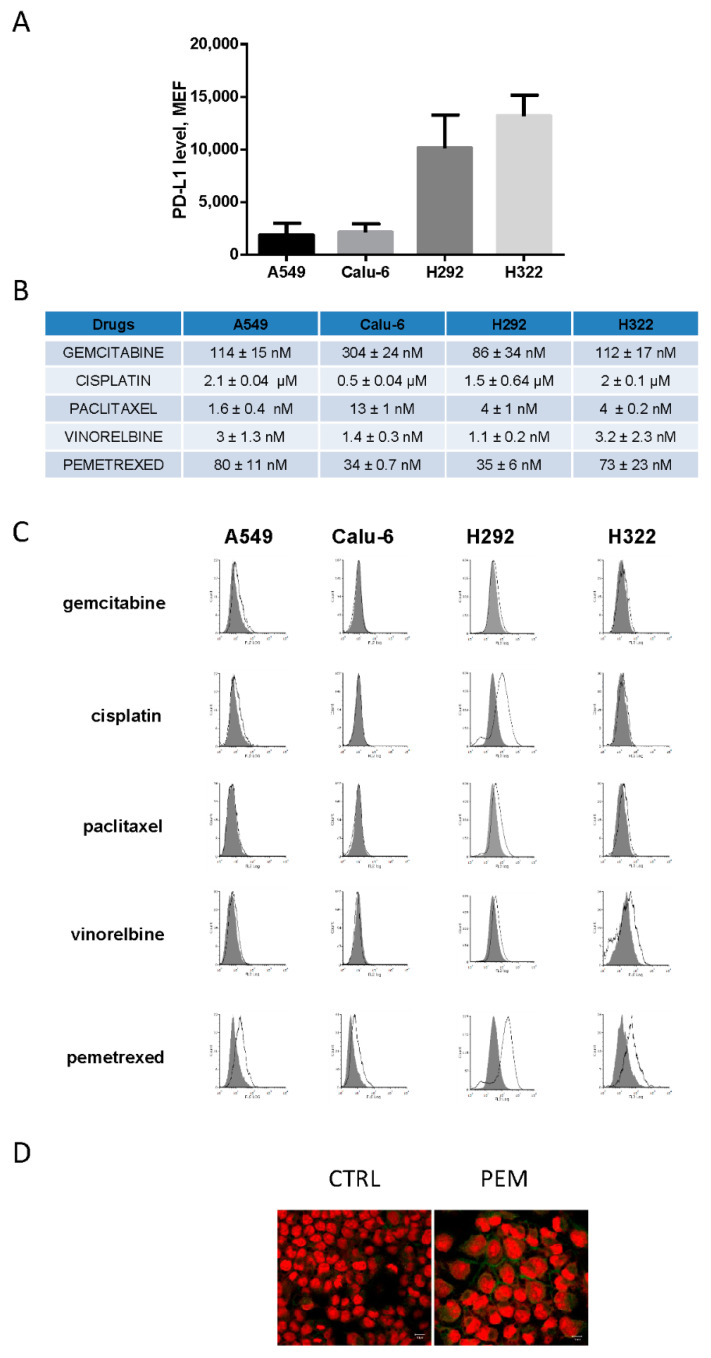
Modulation of programmed cell death ligand-1 (PD-L1) levels by chemotherapeutic drugs in non-small-cell lung cancer (NSCLC) cell lines. (**A**) A549, Calu-6, H292, and H322 cells were cultured in complete medium and after 24 h, the cells were collected and PD-L1 expression was evaluated by flow cytometry and quantified as molecules of equivalent fluorochrome (MEF). (**B**) Cancer cells were treated with increasing concentrations of the indicated drugs for 72 h. IC_50_ values ± SD were calculated using GraphPad Prism 6.00 software. (**C**) NSCLC cells were treated with gemcitabine, cisplatin, paclitaxel, vinorelbine, or pemetrexed at IC_50_ concentrations. After 3 days, PD-L1 levels were analyzed by flow cytometry. (**D**) Confocal immunofluorescence analysis of PD-L1 expression in H322 cells maintained in the absence or in the presence of 100 nM pemetrexed for 72 h; green fluorescence indicates the positivity to PD-L1. The nuclei were stained with Draq5 (red fluorescence). Scale Bar: 10 μm. Data in (**A**) and (**B**) are mean values ± SD of three independent experiments. Results in (**C**) and (**D**) are representative of two independent experiments.

Firstly, we evaluated PD-L1 membrane level (mPD-L1) by flow cytometry in four NSCLC cell lines (A549, Calu-6, H292, and H322) with the non-squamous histotype and wild-type for *EGFR* and *ALK*. As shown in Figure 1A, PD-L1 was expressed at different levels, with a higher expression in H292 and H322 compared to A549 and Calu-6 cells.

The surface expression of PD-L1 in A549 and H322 cells (Figure S1) was then evaluated upon treatment with IFN-γ, a key cytokine known to upregulate PD-L1 at the membrane level [19]. As expected, the addition of IFN-γ significantly increased PD-L1 in both cell lines, indicating the presence of a functional Jak/STAT signaling pathway [20], known to control PD-L1 expression. Then, we tested the effect of different chemotherapeutic drugs (the antimetabolites, gemcitabine and pemetrexed; the microtubule-targeting agents, paclitaxel and vinorelbine; and the DNA-damaging agent, cisplatin) on mPD-L1 expression. A549, Calu-6, H292, and H322 cells were treated with each compound at the corresponding IC_50_ value indicated in Figure 1B. As reported in Figure 1C, pemetrexed caused a marked increase in mPD-L1 expression in all the tested cell lines. In H292 cells, cisplatin increased mPD-L1 expression. Pemetrexed-mediated induction of mPD-L1 expression was confirmed by confocal microscopy in H322 cells, as shown in Figure 1D.

**Figure 2:**
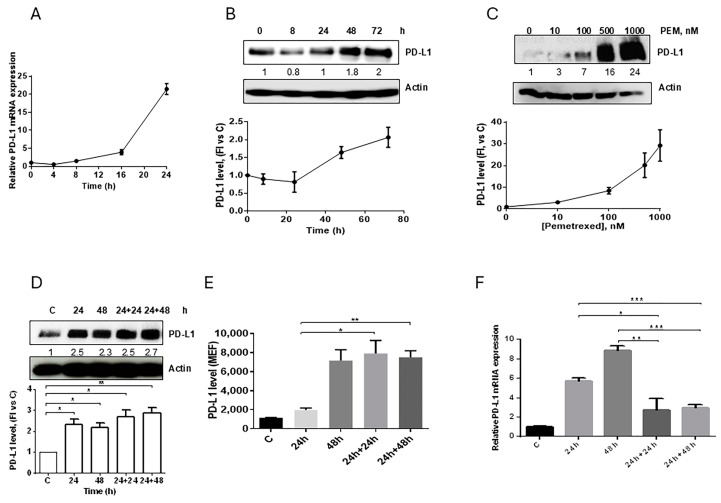
Effect of pemetrexed on PD-L1 expression in A549 cell line. (**A**) A549 cells were treated with 100 nM pemetrexed for the indicated period of time and *PD-L1* mRNA level, evaluated by RT-PCR, was reported. (**B**) Time-dependent modulation (100 nM pemetrexed) and (**C**) dose-dependent modulation (72 h) of PD-L1 protein expression in A549 cells were evaluated by western blotting. A549 cells were continuously exposed to 500 nM pemetrexed for the indicated period of time or treated for 24 h and, after drug removal, the cells were incubated with fresh medium for 24 h or 48 h. At the indicated times, total PD-L1 protein, membrane PD-L1 protein, and *PD-L1* mRNA were quantified by western blotting (**D**), flow cytometry (**E**), and RT-PCR (**F**), respectively. * *p* < 0.05; ** *p* < 0.01; *** *p* < 0.001. Data in (**A**), (**E**), and (**F**) are mean values ± SD of three independent experiments. Results in (**B**–**D**) are representative of three independent experiments.

We then evaluated the effect of pemetrexed at different exposure times and concentrations on PD-L1 expression in A549 cells. Pemetrexed at 100 nM increased *PD-L1* mRNA level (Figure 2A) and protein expression (Figure 2B) in a time-dependent manner with the highest levels of PD-L1 protein detected at 72 h. At this time, we evaluated the effect of increasing concentrations of the drug on PD-L1 induction, demonstrating that PD-L1 level started to increase at 100 nM with the maximum expression observed at 500–1000 nM (Figure 2C). Pemetrexed at 500 nM enhanced PD-L1 level after 24 h (Figure 2D and Figure S2). 

With the aim to evaluate whether the induced PD-L1 expression was also maintained after pemetrexed removal, A549 cells were treated for 24 h with 500 nM of the drug and then incubated in drug-free medium for up to 48 h. Total (Figure 2D) and membrane (Figure 2E) PD-L1 expression were unchanged for 48 h after pemetrexed removal, maintaining levels comparable to those of cells continuously exposed to pemetrexed for 48 h, despite the significant decrease in *PD-L1* mRNA expression (Figure 2F). These results suggest that, upon induction, PD-L1 is a stable protein. 

### 2.2. Pemetrexed-Induced PD-L1 Expression Is Mediated by mTOR and STAT3 Signaling

**Figure 3:**
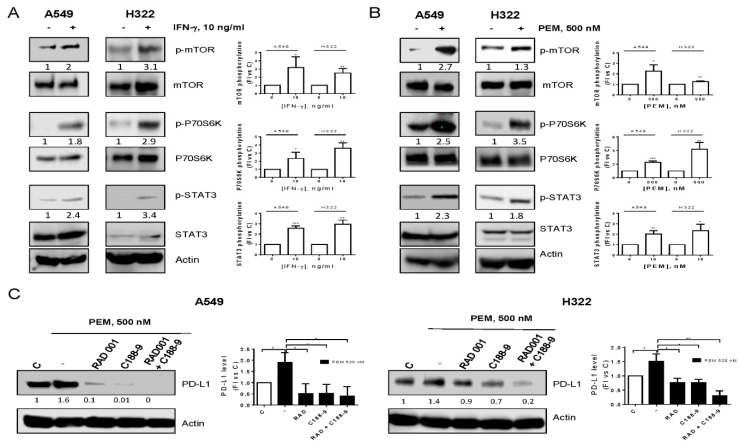
Pemetrexed induced mTOR and STAT3 pathway activation. A549 and H322 cells were treated with 10 ng/mL IFN-γ (**A**) or 500 nM pemetrexed (**B**) for 16 h and cell extracts were analyzed by western blotting with the indicated antibodies. (**C**) A549 and H322 cells were pre-incubated with 100 nM RAD001 or 25 µM C188-9; after 4 h, 500 nM pemetrexed was added to the cells; and 24 h later, cell extracts were analyzed by western blotting. Data are representative of three independent experiments. * *p* < 0.05; ** *p* < 0.01; *** *p* < 0.001.

The regulation of PD-L1 expression is very complex, varying among different tumor types and including both transcriptional and post-transcriptional mechanisms of control [8]. To unravel the mechanisms of pemetrexed-mediated upregulation of PD-L1, we compared the effects of IFN-γ and pemetrexed on intracellular signaling pathways. Results showed that IFN-γ or pemetrexed caused an increase in the phosphorylation of mTOR, P70S6K, and STAT3 proteins in A549 and H322 cells (Figure 3A,B). 

To define the timing of PD-L1 induction with respect to cell signaling modulation, we performed a time-course experiment demonstrating that both mTOR and STAT3 signaling were activated before the induction of PD-L1, at 6 h and 16 h of treatment for mTOR and STAT3 activation, respectively (Figure S2). 

To clarify the role of these signaling cascades on PD-L1 upregulation, treatment with specific inhibitors was performed (Figure 3C). Pre-incubation of A549 and H322 cells with the mTOR inhibitor RAD001 reduced PD-L1 induction by pemetrexed; a more evident reduction was observed after pre-incubation with the STAT3 inhibitor C188-9. Interestingly, when the cells were pre-treated with both compounds, PD-L1 expression was further downregulated, suggesting that both pathways control PD-L1 expression induced by pemetrexed.

### 2.3. Pemetrexed Induces PD-L1 Expression through Stimulation of IFN-γ Production by T Lymphocytes 

Since chemotherapeutic drugs interact not only with cancer cells, but also with immune cells in the tumor microenvironment, we tested the effects of pemetrexed on cancer cells co-cultured with activated PBMCs in a non-contacting transwell system (Figure S3A).

**Figure 4:**
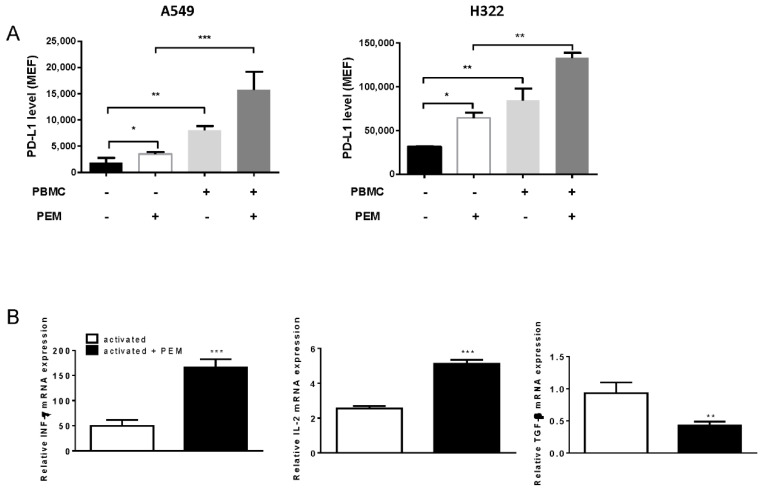
PD-L1 modulation by pemetrexed in a co-culture system. (**A**) A549 and H322 cells were cultured in transwells in the absence or in the presence of pre-activated T-cells, with or without 500 nM pemetrexed for 24 h; at the end of the experiment, the cells were collected and PD-L1 levels were analyzed by flow cytometry and quantified as MEF. (**B**) Isolated PBMCs were activated for 24 h with anti-CD3/CD28 and then treated with pemetrexed 100 nM for 24 h. The cells were lysed and total RNA was extracted. The mRNA levels of INF-ϒ, IL-2 and TGF-β were analyzed by RT-PCR and normalized to quiescent cells. (* *p* < 0.05; ** *p* < 0.01; *** *p* < 0.001). Experiments are the mean of three independent measures.

A549 and H322 cells were co-cultured with primary human T-cells stimulated with CD3/CD28, in the absence or presence of pemetrexed. As reported in Figure 4A, a significant increase of mPD-L1 was observed when activated lymphocytes were added to the upper chamber of the transwell. The addition of pemetrexed to the co-culture system promoted a further increase in mPD-L1 level. 

We then sought to elucidate the mechanisms underneath the effects of pemetrexed treatment on T-cell activation. Pemetrexed strongly enhanced the mRNA expression of *IFN-γ* and *IL-2*, and downregulated *TGF-β* mRNA levels (Figure 4B) in activated T-cells. 

**Figure 5:**
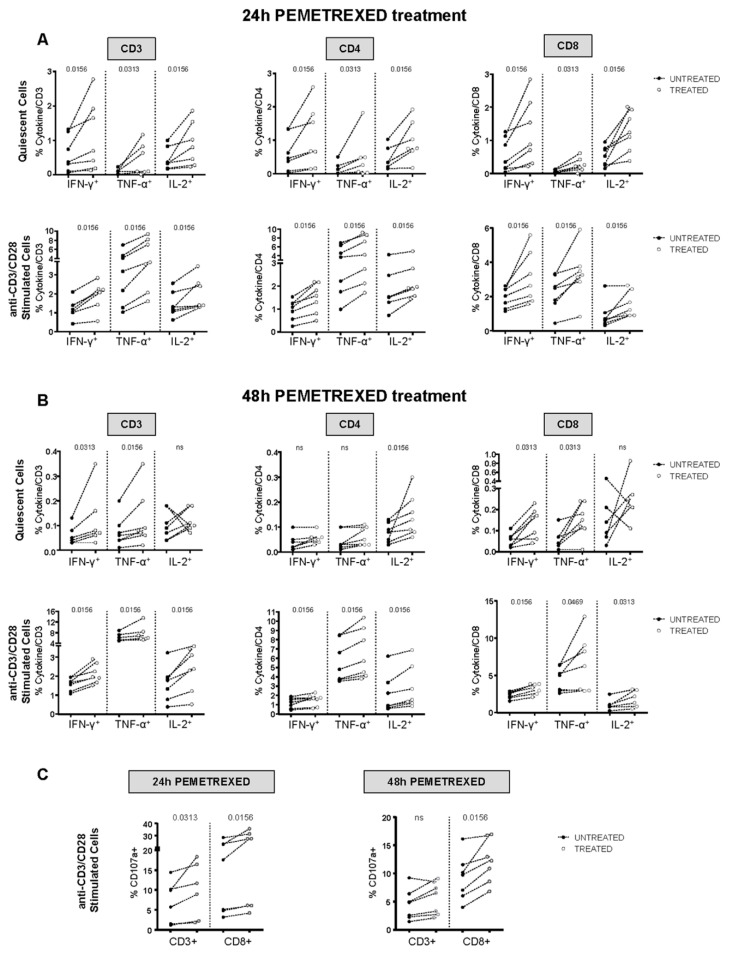
In vitro pemetrexed treatment improved T-cell responses. (**A**) PBMCs from healthy donors (*n* = 7) were stimulated for 24 h with anti-CD3/anti-CD28 antibodies in the presence or absence of 100 nM pemetrexed. This was followed by co-staining for CD3, CD4, or CD8 plus IFN-γ, TNF-α, or IL-2 to determine T-cell function by flow cytometry. Upper and lower plots show results from unstimulated and stimulated PBMCs, respectively, with and without 24 h pemetrexed treatment. Data are presented as cytokine-positive, CD3+/CD4+/CD8+ T-cells detected in treated and untreated cultures, white and black dots, respectively. (**B**) PBMCs from healthy donors (*n* = 7) were stimulated for 48 h as in (**A**). (**C**) PBMCs from healthy donors (*n* = 6) were stimulated for 24 and 48 h as in (**A**) together with CD107a antibody to evaluate the cytotoxic potential by flow cytometry. Data are presented as CD107a-positive, CD3+, and CD8+ T-cells detected in treated and untreated cultures, white and black dots, respectively. (**A**–**C**) All data were analyzed with the Kolmogorov–Smirnov test to confirm or exclude a normal distribution. A statistical analysis was done by two-tailed Wilcoxon matched-pairs test and *p*-values are indicated.

We further investigated pemetrexed-induced cytokine production by quiescent and anti-CD3/CD28-activated T cells in vitro. Treatment with pemetrexed enhanced IFN-γ, TNF-α, and IL-2 production in both quiescent and activated cells after 24 h of treatment (Figure 5). Of note, the same significant trend was evident for CD3-, CD4-, and CD8-positive T cells (Figure 5A). However, after 48 h of pemetrexed treatment, a significant increase in cytokine production was observed only in anti-CD3/CD28-stimulated T cells (Figure 5B). In addition, the cytotoxic potential was augmented after 24 h of pemetrexed treatment as compared to 48 h (Figure 5C), predominantly for CD8-positive T cells. Then, we investigated the effect of paclitaxel and cisplatin on T-cell responses in quiescent and stimulated cells, and demonstrated that neither of these drugs affected cytokine production and the cytotoxic potential (Figures S5A,B and S6A,B). Of note, all the chemotherapeutic treatments induced only a slight increase in cell death in lymphocytes (Figure S4). 

In conclusion, these findings suggest that pemetrexed can affect the expression of PD-L1 on cancer cells either directly, by activating signaling pathways controlling PD-L1 transcription, or indirectly, through the release of IFN-γ by immune cells in the tumor microenvironment.

### 2.4. Effect of Pemetrexed Treatment on the Release of Soluble Form of PD-L1 

**Figure 6:**
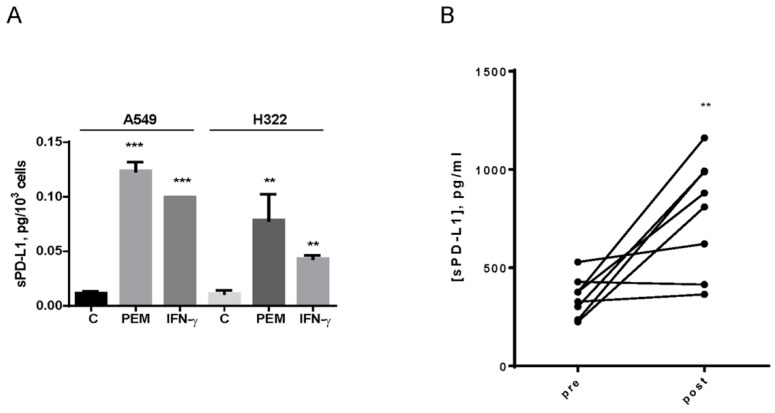
sPD-L1 levels in cell culture medium and in plasma from NSCLC patients treated with pemetrexed. (**A**) A549 and H322 cells were treated with 100 nM pemetrexed for 3 days; at the end, sPD-L1 was quantified by ELISA assay in the culture medium and data were normalized to cell number. IFN-γ at 10 ng/mL was used as an internal control. Data are representative of three independent experiments. (**B**) sPD-L1 detected in plasma from 8 NSCLC patients by ELISA assay before and after 4 days of pemetrexed treatment. ** *p* < 0.01; *** *p* < 0.001.

Besides the membrane-bound form, PD-L1 has a soluble form (sPD-L1) that is released in the extracellular milieu. Previous data indicated that cancer cell lines with high mPD-L1 present elevated PD-L1 protein levels in the supernatant [21]. It is also well known that cytokines, such as IFN-γ, can increase the release of sPD-L1 [22]. We then evaluated if sPD-L1 can be released in response to pemetrexed treatment. Pemetrexed, similar to IFN-γ, not only increased mPD-L1, but also induced the release and accumulation of sPD-L1 in the medium from both A549 and H322 cells (Figure 6A).

To evaluate whether our preclinical data could be translated into a clinical setting, we assessed sPD-L1 levels in plasma samples from eight NSCLC patients, collected 4 days after pemetrexed treatment. As reported in Figure 6B, this drug significantly increased sPD-L1 levels in patients.

### 2.5. The Addition of the Anti PD-L1 Antibody Atezolizumab to Pemetrexed Significantly Potentiates T cell-Mediated Cytotoxicity in NSCLC Cells

Finally, we investigated in a co-culture assay, the anti-tumor immune activity of atezolizumab (anti PD-L1) with or without pemetrexed in the presence of activated T-cells.

**Figure 7:**
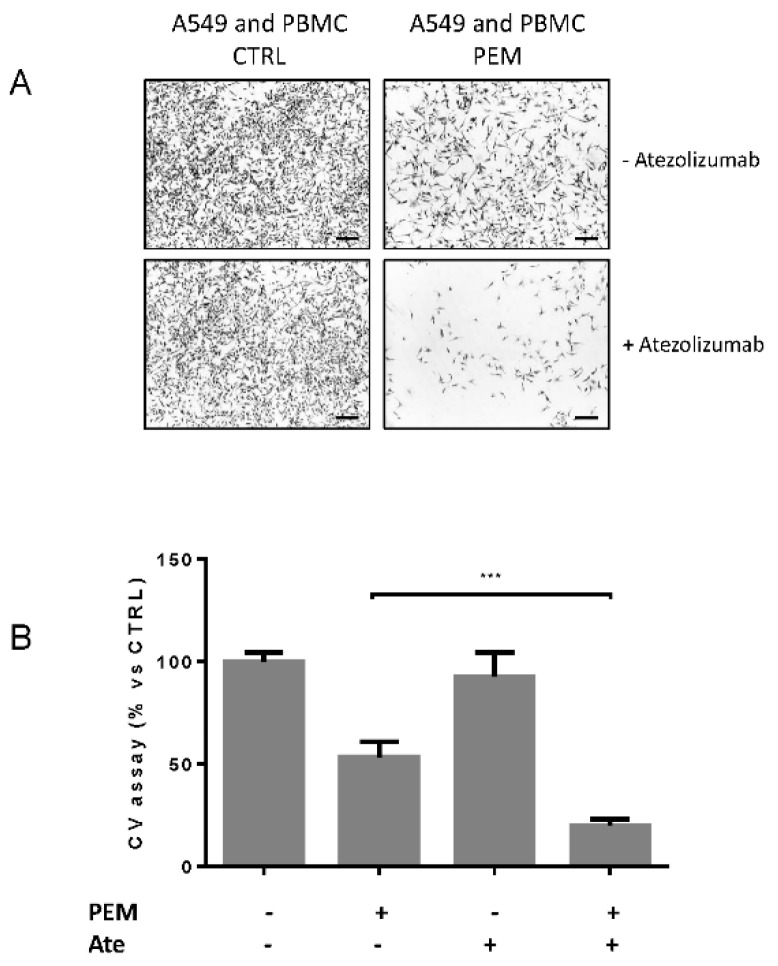
Atezolizumab increased T cell-mediated cytotoxicity in A549 cells treated with pemetrexed. A549 tumor cells and PBMCs activated with anti-CD3 (1 μg/mL) and anti-CD28 (2 μg/mL) were co-cultured in transwells for 3 days with or without 100 nM pemetrexed. Then PBMC were transferred to contacting co-culture by transferring them from the insert to the corresponding well with a ratio of approximately 5:1 (effector: target cells) for 24 h with or without 200 µg/mL atezolizumab. (**A**) Images of A549 cells co-cultured with PBMCs in the absence or presence of 100 nM pemetrexed with or without 200 µg/mL atezolizumab. Scale bar: 100 μm. (**B**) Crystal violet assay *** *p* < 0.01. Data in (**B**) are the average of three independent experiments.

For this purpose, A549 cells were co-cultured in a transwell system (non-contacting co-culture) with activated PBMCs in the presence or absence of 100 nM pemetrexed for 3 days (Figure S3A). Then, activated PBMCs were transferred from the insert to the corresponding well (Figure S3B) to be in contact with A549 cells (Figure S3C). The contacting co-culture was then treated for 24 h with or without 200 µg/mL atezolizumab. Phase contrast microscopy images (Figure 7A) and crystal violet assay (Figure 7B) showed that atezolizumab significantly promoted T cell-mediated cytotoxicity of A549 cells treated with pemetrexed. These data suggest that PD-L1 expression induced by pemetrexed is important for atezolizumab to enhance T cell-mediated cytotoxicity in NSCLC cells.

## 3. Discussion

In this work, we demonstrated that pemetrexed has the ability to stimulate PD-L1 expression in NSCLC cells, either as a membrane-bound protein or as a released soluble form. Moreover, pemetrexed caused a marked increase in the production of pro-inflammatory cytokines, including IFN-γ, by T cells, which further enhanced PD-L1 expression on cancer cells. This increased level strongly enhanced T cell-mediated cytotoxicity induced by anti-PD-L1 therapy, as evaluated in co-culture systems. 

Since the approval of the anti-PD-1 monoclonal antibodies nivolumab and pembrolizumab and the anti-PD-L1 inhibitor atezolizumab for the treatment of advanced NSCLC, new strategies have been developed in order to further improve the efficacy of these therapies. Recently, the phase III clinical trial Keynote-189 [4] showed that pembrolizumab combined with the pemetrexed-platinum standard chemotherapy regimen significantly increased overall survival in advanced non-squamous NSCLC patients with different levels of PD-L1 expression. Interestingly, the results of this clinical trial showed that immunotherapy was able to improve chemotherapy outcome regardless of baseline PD-L1 expression. In particular, a subgroup analysis showed no correlation between PD-L1 expression and the amount of benefit derived from the addition of pembrolizumab to platinum-pemetrexed chemotherapy and, surprisingly, even patients with null PD-L1 expression received a statistically and clinically relevant benefit from the combined treatment as those with different levels of PD-L1 expression. One of the likely explanations for this unexpected clinical finding may be related with the possible modulation of PD-L1 during chemotherapy treatment. 

Very recently, it has been reported [7] that the PD-L1 tumor proportion score was much higher in re-biopsy tissue samples when compared with the initial biopsy in some NSCLC patients after chemotherapeutic treatment. A clinical trial (NCT03701607) aiming to evaluate whether PD-L1 may change after platinum-based chemotherapy in advanced NSCLC is presently active for patient recruitment.

In our study performed in a panel of NSCLC adenocarcinoma cell lines, wild-type for *EGFR* and *ALK*, we demonstrated that PD-L1 levels were enhanced after treatment with pemetrexed, but not with other chemotherapeutic agents, as a consequence of the activation of mTOR/P70S6K and STAT3 intracellular signaling pathways. Although the ability of pemetrexed to induce mTOR activation has already been reported [23], here we demonstrated that pemetrexed also activated STAT3, the main transcription factor responsible for PD-L1 activation mediated by IFN-γ [24]. According to this observation, pharmacological inhibition of both the signaling pathways with RAD001 and C188-9 reduced PD-L1 levels in cancer cells. Pemetrexed exposure also caused an increase in the soluble form of PD-L1. Recent data described discordant results on the predictive and prognostic significance of sPD-L1 in lung cancer patients [25], and the ability of sPD-L1 to interact with its ligand PD-1 on T cells is still on debate. Nevertheless, PD-L1 plasma levels were significantly enhanced in NSCLC patients receiving standard first-line chemotherapy [8] and our data indicated that pemetrexed, as a single regimen, can increase sPD-L1 in plasma patients.

In activated PBMCs, pemetrexed induced a marked increase in the expression and release of the cytokines IFN-γ and IL-2, and a decrease of the anti-inflammatory cytokine TGF-β, a sign of the acquisition of a pro-inflamed phenotype. Similar results were observed in patients with adenocarcinoma of the pancreas treated with the anti-folate compound; the authors reported an increase of IFN-γ and IL-2 secretion by natural killer (NK) cells during the treatment, enforcing the role of pemetrexed as a drug that can alter cytokine production in cancer patients [26].

The secretion of IFN-γ by T lymphocytes increased the expression of PD-L1 on cancer cells, thereby enhancing the efficacy of the immune checkpoint inhibitor atezolizumab, as demonstrated in the contacting co-culture assay, thus suggesting that a high PD-L1 level can be considered as a “driver” for T cell-mediated anti-tumor activity of immune checkpoint inhibitors.

These results are in agreement with recent findings showing that pemetrexed treatment in murine syngeneic colon tumor models (MC38 and Colon 26) activated T cells, inducing an inflamed phenotype, and that the addiction of an anti PD-L1 antibody improved the anti-tumor properties of the anti-folate compound [27].

Most chemotherapeutic drugs have been shown to have a detrimental effect on CD8+ T-cells in lymph nodes and blood; however, the same effect has not been detected on tumor-infiltrating lymphocytes [28]. Conventional chemotherapy can instead increase anti-tumor responses through the induction of immunogenic cell death by depleting immunosuppressive immune cell subsets, such as myeloid-derived suppressor cells or regulatory T-cells, or by exerting direct stimulatory effects on immune cells by cytokine production [29,30]. For example, cisplatin and paclitaxel treatment has been reported to generate a marked CD8+ T-cell response with high secretion of IFN-γ and IL-2 in patients with ovarian cancer [31]; 5-FU or gemcitabine selectively killed myeloid-derived suppressor cells [32] and in patients with esophageal squamous cell carcinoma, adjuvant chemotherapy with cisplatin and 5-FU increased the trafficking of CD4+ and CD8+ cells in the tumor microenvironment [33].

In conclusion, we propose that pemetrexed, by promoting PD-L1 upregulation in tumor cells and by inducing the secretion of cytokines by T cells, may generate, in the tumor microenvironment, a favorable condition for the efficacy of immune checkpoint inhibitors, independently of PD-L1 tumor cell expression at baseline.

## 4. Materials and Methods 

### 4.1. Cells and Cell Culture

Human NSCLC A549, Calu-6, H292, and H322 cell lines were purchased from the American Type Culture Collection (ATCC, Rockville, MD, USA) and cultured in Roswell Park Memorial Institute medium (RPMI 1640) supplemented with 2 mM glutamine, 10% fetal bovine serum (FBS), 100 U/mL penicillin, and 100 µg/mL streptomycin. The cell lines were incubated at 37 °C in a humidified atmosphere containing 5% CO_2_. 

### 4.2. Reagents

Pemetrexed, cisplatin, gemcitabine, paclitaxel, vinorelbine, and atezolizumab were obtained from the Inpatient Pharmacy of University Hospital of Parma. RAD001 and the STAT3 inhibitor C188-9 were from Selleck Biochemicals (Houston, TX, USA). Human recombinant IFN-γ was from ImmunoTools (Friesoythe, Germany).

### 4.3. Flow Cytometry 

For the determination of membrane levels of PD-L1, cells were treated for 3 days with chemotherapeutic drugs, collected, and incubated with phycoerythrin (PE) isotype control mouse IgG1 κ or PE anti-human PD-L1 (BD Biosciences, San Jose, CA, USA). After incubation, quantification was performed with a Beckman FC500 flow cytometer as previously described [34] and analyzed with FCS express software (De Novo software, Pasadena, CA, USA). The values of mean fluorescence intensity (MFI) were converted into units of equivalent fluorochrome (MEF) using the FluoroSpheres 6-Peak kit (Dako, Santa Clara, CA, USA).

### 4.4. Cell Proliferation Assay 

Cell proliferation was evaluated by cell counting, the MTT assay, and crystal violet staining as previously described [35].

### 4.5. Confocal Microscopy

Cells were grown on poly-L-lysine-coated glass slides and treated with 100 nM pemetrexed. After 72 h, the cells were fixed with 4% paraformaldehyde and permeabilized with 0.2% Triton X-100, and unspecific epitopes were blocked with 2% bovine serum albumin (BSA). Then, for PD-L1 staining, the cells were incubated overnight at 4 °C with anti-PD-L1 (Abcam, ab205921) and Alexa Fluor 546-conjugated donkey anti-rabbit IgG (Invitrogen, Carlsband, CA, USA) was used as the secondary antibody. Nuclei were stained with Draq5 (Biostatus, Shepshed, Leicestershire, UK). Samples were observed using a confocal system (LSM 510 Meta, scan head integrated with the Axiovert 200 M inverted microscope; Carl Zeiss, Jena, Germany) through a 40×, NA 1.3, oil immersion objective. Alexa Fluor 546 and Draq5 were excited with 543 nm and 633 nm He-Ne laser lines, respectively. Image acquisition was carried out in a multitrack mode (through consecutive and independent optical pathways) with relevant beam splitters; barriers filters were 560–615 band pass and 650 long pass filters for the above signals, respectively. 

### 4.6. Western Blotting

Western blotting was performed as previously described [34]. Antibodies against PD-L1, p-mTOR (Ser2448), mTOR, p-P70S6K (Thr389), P70S6K, p-STAT3 (Tyr705), and STAT3 were from Cell Signaling Technology Inc. (Beverly, MA, USA) and anti-β-actin (clone B11V08) was from BioVision (Milpitas, CA, USA). Primary antibodies were diluted 1:1000 in Tween-Tris Buffered Saline TTBS with 5% milk of BSA. The chemiluminescent signal was acquired by the C-DiGit® Blot Scanner and the spots were quantified by the Image Studio™ Software (LI-COR Biotechnology, Lincoln, NE, USA).

### 4.7. Isolation of PBMCs and T-cell Activation

Human PBMCs were isolated from the buffy coat of healthy donors by using the Lympholyte-H density gradient centrifugation media (Cederlane Burlington, Canada) as previously described [36]. PBMCs were activated with soluble anti-CD3 (1 µg/mL) and anti-CD28 (2 µg/mL) (BioLegend, San Diego, CA, USA) for 48 h [37].

### 4.8. PBMC Cytokine Production

To evaluate IFN-γ, TNF-α, and IL-2 production, PBMCs were stimulated for 24 or 48 h with soluble anti-CD3 (1 μg/mL) and anti-CD28 (2 μg/mL) (BioLegend), in the presence or absence of pemetrexed, cisplatin, or paclitaxel. To analyze the cytotoxic potential, CD107a PE-Vio770 (Miltenyi Biotec, Bergisch Gladbach, Germany) was added to the cells in the last 18 h of stimulation. To block cytokine secretion, the Golgi inhibitor brefeldin A (10 mg/mL, Sigma-Aldrich, St. Louis, MO, USA) was added for 4 h or 18 h to evaluate T-cell responses after 24 h or 48 h of stimulation, respectively. After surface staining with anti-CD3 PECF594 (BD Biosceinces, San Jose, CA, USA), CD8-PerCP (Miltenyi Biotec), or CD4-PE (Miltenyi Biotec), cells were fixed with the medium A reagent (Nordic Mubio, Susteren, The Netherlands). Then, cells were permeabilized with the medium B reagent (Nordic Mubio) and stained together with anti-IFN-γ APC-R700 (BD Biosciences), anti-TNF-α FITC (Miltenyi Biotec), and anti-IL2 APC (BD Biosciences) monoclonal antibodies for intracellular cytokine staining, in accordance with the manufacturers’ instructions. Cytokine production was quantified by flow cytometry (FACS Canto II, BD Biosciences) and analyzed with the FacsDIVA Software (BD BIosciences). In parallel, to investigate T-cell death during 24 h and 48 h stimulations, cells were stained with anti-CD3-FITC (BioLegend), anti-CD8-APC-H7 (BD Biosciences), anti-CD4-PE (Miltenyi Biotec), and the vitality dye 7AAD (BD Biosciences).

### 4.9. Quantitative Real-Time PCR

Total RNA was isolated by the RNAeasy Mini Kit (Qiagen, Venlo, The Netherlands) and quantitative real-time polymerase chain reaction (PCR) was performed as previously described [38]. Primers for hPD-L1 (QT0082775), hIL-2 (QT00089964) hINF-γ (QT00059066), and hTGF-β (QT00001302) were purchased from Qiagen.

### 4.10. Soluble PD-L1 Quantification

To assay soluble PD-L1 (sPD-L1), cells were seeded in a cell-well culture plate. After 24 h, pemetrexed or IFN-γ was added for 3 days; at the end, the media were collected and tested for soluble PD-L1 expression by ELISA assay (R&D System, Minneapolis, MN, USA).

### 4.11. Co-Culture System

A non-contacting co-culture system of cancer cells with PBMCs was established using a transwell suspension culture chamber with a polyethylene terephthalate film (PET) (Corning, NY, USA). Briefly, 1 × 10^5^ cells were seeded in a six-well plate. At the same time, freshly isolated PBMCs were activated with CD3/CD28. The day after, 1 × 10^6^ activated PBMCs were added to the transwell in the presence or absence of pemetrexed and after 24 h, PBMCs were removed and PD-L1 membrane levels on cancer cells were analyzed by a flow cytometer.

For T cell-mediated cytotoxicity [39,40], after 3 days of incubation in the previous reported transwell system, PBMCs were transferred from the insert to the corresponding well in a contacting co-culture with A549 cells and maintained for 24 h in the absence or presence of 200 µg/mL atezolizumab. Finally, PBMCs and dead tumor cells were washed away and the crystal violet assay was performed. Images of viable cells were obtained with a Nikon Eclipse E400 microscope with a digital net camera.

### 4.12. Patients

Plasma samples were obtained from NSCLC patients enrolled within a phase Ib trial of pemetrexed-enzastaurin combination therapy, as described previously [41]. All patients received standard pemetrexed as a 10 min intravenous dose of 500 mg/m^2^. All these patients also received oral folic acid (350–1000 μg daily) and vitamin B12 (1000 μg by intramuscular injection every 9 weeks), beginning 5–7 days before the first dose of pemetrexed. An analysis of soluble PD-L1 expression was performed by ELISA assay (R&D System, Minneapolis, MN, USA).

### 4.13. Statistical Analysis

Statistical analyses were carried out using the GraphPad Prism 6.00 software. The normality distribution of data was tested by the Kolmogorov–Smirnov test. Comparisons were performed by the two-tailed Wilcoxon matched-pairs test, two-tailed Student’s t-test, or one-way ANOVA followed by Bonferroni’s post-test, depending on the assay; and *p*-values are indicated where appropriate (* *p* < 0.05, ** *p* < 0.01, *** *p* < 0.001, **** *p* < 0.0001). 

## 5. Conclusions

Our findings provide a biological rationale to explain the superior efficacy of anti-PD1/PD-L1 therapy added to platinum-pemetrexed chemotherapy in patients with PD-L1-negative NSCLC and support pemetrexed as an ideal drug partner for chemoimmunotherapy combination regimens in advanced non-squamous NSCLC treatment. The optimal pemetrexed scheduling along with immune checkpoint inhibitors, concurrent vs sequential, in order to take maximum advantage of the interaction between chemotherapy and PD-L1 expression, remains to be elucidated and deserves further pre-clinical and clinical research.

## Supplementary Materials

The following are available online at http://www.mdpi.com/2072-6694/12/3/666/s1, Figure S1: PD-L1 modulation by IFN-γ in NSCLC cell lines, Figure S2: PD-L1 induction by mTOR and STAT3 signaling activation, Figure S3: Tumor cells and PBMC non-contacting and contacting co-culture, Figure S4: Pemetrexed/cisplatin/paclitaxel-induced cell death on stimulated and unstimulated T-cells, Figure S5: In vitro effect of 24 h cisplatin and paclitaxel treatments on stimulated and unstimulated T-cell responses, Figure S6: In vitro effect of 48 h cisplatin and paclitaxel treatments on stimulated and unstimulated T-cell response.
