# Correction: Cavazzoni et al. Pemetrexed Enhances Membrane PD-L1 Expression and Potentiates T Cell-Mediated Cytotoxicity by Anti-PD-L1 Antibody Therapy in Non-Small-Cell Lung Cancer. *Cancers* 2020, *12*, 666

**DOI:** 10.3390/cancers17081254

**Published:** 2025-04-08

**Authors:** Andrea Cavazzoni, Graziana Digiacomo, Roberta Alfieri, Silvia La Monica, Claudia Fumarola, Maricla Galetti, Mara Bonelli, Daniele Cretella, Valeria Barili, Alessandra Zecca, Elisa Giovannetti, Michelangelo Fiorentino, Marcello Tiseo, Pier Giorgio Petronini, Andrea Ardizzoni

**Affiliations:** 1 Department of Medicine and Surgery, University of Parma, 43126 Parma, Italy; graziana.digiacomo@unipr.it (G.D.); silvia.lamonica@unipr.it (S.L.M.); claudia.fumarola@unipr.it (C.F.); mara.bonelli@unipr.it (M.B.); daniele.cretella@unipr.it (D.C.); barili.valeria@gmail.com (V.B.); marcello.tiseo@unipr.it (M.T.); piergiorgio.petronini@unipr.it (P.G.P.); 2 Italian Workers’ Compensation Authority (INAIL) Research Center, 43126 Parma, Italy; m.galetti@inail.it; 3 Department of Infectious Diseases and Hepatology, University Hospital of Parma, 43126 Parma, Italy; alessandrazecca.az@gmail.com; 4 Department of Medical Oncology, Cancer Center Amsterdam, Amsterdam University Medical Center, 1081HV Amsterdam, The Netherlands; elisa.giovannetti@gmail.com; 5 Fondazione Pisana per la Scienza, San Giuliano Terme, 56017 Pisa, Italy; 6 Department of Experimental, Diagnostic and Specialty Medicine, University of Bologna, 40138 Bologna, Italy; michelangelo.fiorentino@unibo.it (M.F.); andrea.ardizzoni2@unibo.it (A.A.)

## Error in Figure

In the original publication [1], there was a mistake in Figure 2C,D as published. An incorrect image of Actin was inserted as a loading control. The corrected Figure 2 appears below. Additionally, the associated Supplementary Figure S7 has been updated. The authors apologize for any inconvenience caused and state that the scientific conclusions are unaffected. This correction was approved by the Academic Editor. The original publication has also been updated.

**Figure 2:**
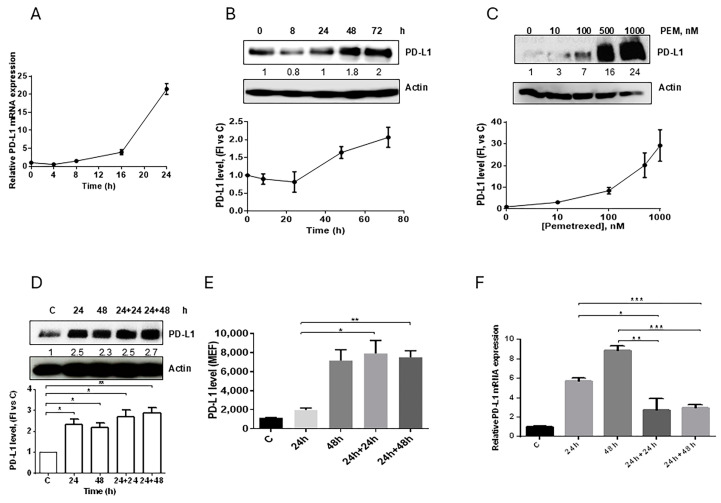
Effect of pemetrexed on PD-L1 expression in A549 cell line. (**A**) A549 cells were treated with 100 nM pemetrexed for the indicated period of time and *PD-L1* mRNA level, evaluated by RT-PCR, was reported. (**B**) Time-dependent modulation (100 nM pemetrexed) and (**C**) dose-dependent modulation (72 h) of PD-L1 protein expression in A549 cells were evaluated by western blotting. A549 cells were continuously exposed to 500 nM pemetrexed for the indicated period of time or treated for 24 h and, after drug removal, the cells were incubated with fresh medium for 24 h or 48 h. At the indicated times, total PD-L1 protein, membrane PD-L1 protein, and *PD-L1* mRNA were quantified by western blotting (**D**), flow cytometry (**E**), and RT-PCR (**F**), respectively. * *p* < 0.05; ** *p* < 0.01; *** *p* < 0.001. Data in (**A**), (**E**), and (**F**) are mean values ± SD of three independent experiments. Results in (**B**–**D**) are representative of three independent experiments.
